# Influence of Inflammation on Poststroke Plasticity

**DOI:** 10.1155/2013/258582

**Published:** 2013-02-21

**Authors:** Monika Liguz-Lecznar, Malgorzata Kossut

**Affiliations:** ^1^Laboratory of Neuroplasticity, Nencki Institute of Experimental Biology, Polish Academy of Science, 3 Pasteur Street, 02-093 Warsaw, Poland; ^2^Warsaw School of Social Science and Humanities, 19 Chodakowska Street, 03-815 Warsaw, Poland

## Abstract

Age-related brain injuries including stroke are a leading cause of morbidity and mental disability worldwide. Most patients who survive stroke experience some degree of recovery. The restoration of lost functions can be explained by neuronal plasticity, understood as brain ability to reorganize and remodel itself in response to changed environmental requirements. However, stroke triggers a cascade of events which may prevent the normal development of the plastic changes. One of them may be inflammatory response initiated immediately after stroke, which has been found to contribute to neuronal injury. Some recent evidence though has suggested that inflammatory reaction can be also neuroprotective. This paper attempts to discuss the influence of poststroke inflammatory response on brain plasticity and stroke outcome. We also describe the recent anti-inflammatory strategies that have been effective for recovery in experimental stroke.

## 1. Introduction

Ischemic stroke results from two main pathological processes: a loss of oxygen and an interruption of glucose supply to a particular brain region. The collapse of energy provision leads to the dysfunction of ionic pumps, loss of membrane potential, and uncontrolled release of neurotransmitters. The consequence of those processes is the increase of intracellular calcium concentrations that, among many deleterious effects, result in the generation of free radicals, leading to disintegration of cell membranes and subsequent neuronal death in the core of infarction [1]. Necrosis in the center of infarction can start a few minutes after stroke and is followed by peri-infarct depolarizations, excitotoxicity, edema, and oxidative stress [2]. 

The more delayed processes accompanying stroke are inflammation and apoptosis. They are initiated several hours after ischemic attack and can persist even for several weeks [3]. 

**Figure 1:**
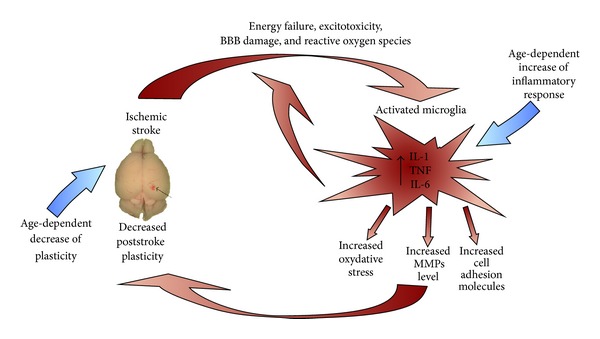
Acute cerebral ischemia, neuroinflammation, and plasticity.

Although a great progress has been made in understanding the cellular and molecular mechanisms of ischemic tissue damage, the only approved therapy is still thrombolysis achieved by intravenous administration of recombinant tissue plasminogen activator (tPA). Unfortunately, short therapeutic window for this therapy strongly limits the fraction of patient that can benefit from the treatment. Moreover, stroke induces a complex cascade of inflammatory response which contributes to the postischemic damage. The complex nature of phenomena after ischemic event hampers a successful design of effective therapeutic strategies (Figure 1). 

Especially desirable are neuroprotective and proregenerative treatments that could support the poststroke recovery. Several animal and human studies revealed that recovery after stroke and restoration of lost functions can be explained by neuronal plasticity, understood as brain ability to reorganize and remodel itself in response to changed environmental requirements [4–6]. Duncan has pointed to the careful defying of recovery, since more stroke survivors will achieve the positive outcome if recovery is defined in terms of disability than if impairments are used to define it [6].

Ischemic stroke triggers an inflammatory cascade via the activation of different molecular mediators. Failure of delivery of energy metabolites leads to the accumulation of glutamate in extracellular space and excitotoxicity. Within the ischemic tissue a reactive oxygen species are generated and integration of brain-blood barrier is disrupted. Microglia are the first nonneuronal cells that respond to injury, and they are the main source of proinflammatory cytokines and chemokines. Their release results in the activation of resident microglia, upregulation of cell adhesion molecules, and mobilization of leukocytes. Increased oxidative stress and cytokine activation contribute to further exacerbation of the inflammatory process including the upregulation of matrix metalloproteinases (MMPs) from astrocytes and microglia that leads to blood-brain barrier (BBB) dysfunction and finally to neuronal cell death. Aging, which is a risk factor for the stroke, further exacerbates the neuroinflammatory pathways and in the same time decreases the potential of neurons for functional plasticity in healthy brain. Since the poststroke recovery and restoration of lost functions can be explained by neuronal plasticity, the diminished ability of reorganization may be the significant factor resulting in poorer functional outcome in elderly stroke patients.

## 2. Plasticity after Stroke: Enhanced or Impaired?

Despite the nonpermissive environment, in most cases of stroke-induced brain damage, some degree of spontaneous recovery can be observed within the first posttrauma months [7, 8]. To address this issue we investigated the spontaneous plasticity of cortical somatosensory representations following a focal photothrombotic unilateral stroke in the barrel cortex of rats to define the reorganization of cortical activity which correlated with poststroke compensatory plasticity. An evolution of the pattern of brain activation in response to stimulation of vibrissae projecting to the damaged barrel cortex was observed through 2 poststroke months. After general loss of metabolic activation in the lesioned hemisphere, we observed a significant increase of activation in the ipsilesional somatosensory areas. Finally, two months after the stroke, the enhanced activation of ipsilateral hemisphere disappeared and in the stroked hemisphere three sites in the undamaged regions of somatosensory cortex: anterior vibrissae, front paw, and hind paw representations, responded to vibrissal input. The appearance of new activation foci correlated with the full recovery of the behavioral functions measured with the gap-crossing test [9]. Similar pattern of changes was reported in human longitudinal studies of poststroke recovery [10]. The studies of Nudo et al. [11] demonstrated that in adult primates cortex the rehabilitation is important for plasticity leading to the reconstruction of injured cortical motor representation. Thus, the poststroke plasticity is use dependent. It has been attributed to activation of existing, but weak connections, axonal outgrowth, dendritic arborizations, and synaptogenesis: phenomena that accompany functional restoration of neuronal networks [5, 10, 12, 13]. Experimental stroke in rats was shown to induce a process of axonal sprouting in peri-infarct tissue [14, 15] that is accompanied by a unique expression pattern of growth-promoting genes and elimination of chondroitin sulfate proteoglycans, which limit axonal sprouting in glial scar adjacent to infarct [16]. Rapid changes in the number length and turnover of dendritic spines in mice can be observed as soon as few minutes after focal ischemia [17], but that initial loss is then followed by dynamic reestablishment of spine synapses [18, 19]. Also it has been shown in experimental stroke [20] and confirmed in human [21] that cortical responsiveness to the impaired extremity after initial loss is gradually restored predominantly in peri-infarct, but also in more distant regions and homotopic sites in the intact hemisphere.

In addition to neuronal death in the ischemic core, the postischemic loss of function is determined by not only the dysfunction of cells in the surrounding penumbra region, but also loss of connections and altered neuronal transmission in more remote brain areas [10, 22, 23]. Thus, compensation for a functional loss should include unmasking the silent pathways and synapses or recruitment of alternative pathways. The second would require axonal sprouting and formations of synapses de novo. Several animal studies demonstrated a long-range sprouting of axons in several different brain connections, including corticocortical, corticospinal, and corticobulbar projections after focal stroke [24–26]. In human, recent studies that used diffusion tensor-imaging technique (DTI) have demonstrated that changes in white matter integrity may be important for recovery of motor function after ischemia [27, 28]. A study in aphasic patients, since aphasia often occurs as the result of stroke, found plasticity of arcuate fasciculus after long-lasting speech therapy [29].

Glial cells can also contribute to poststroke recovery. Astrocytes that infiltrate the area surrounding the ischemic core also support the neuronal regeneration through delivery of trophic factors and lipids. They form the glial scar that embraces the infarction and restricts the range of inflammation [30]. It was shown in different stroke models that in the peri-infarct area the chondroitin sulfate proteoglycans, such as aggrecan, phosphacan, and versican, are reduced as are reduced perineuronal nets formed by those proteoglycans [16, 31]. Such changes may promote plastic modification of neuronal connectivity.

**Figure 2:**
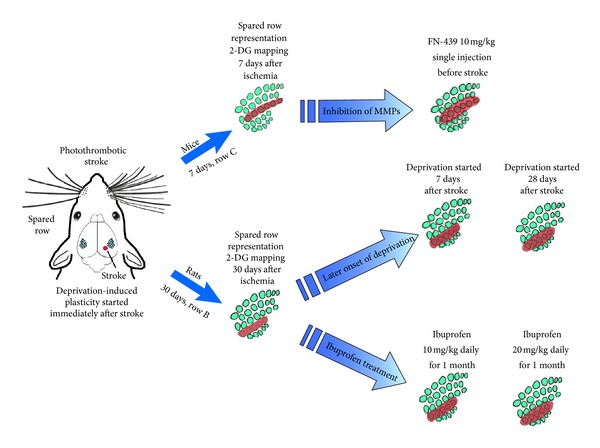
Impairment and restoration of poststroke use-dependent plasticity.

Regardless of the abovementioned observations suggesting the facilitation of plasticity after stroke, other studies brought contradictory results. Using [14C]2-deoxyglucose autoradiography Dietrich and colleagues have described persisting for two months significant decrease of cerebral metabolic rates of glucose throughout the traumatized hemisphere in rats as soon as 4 h after traumatic brain injury. The change was most pronounced in regions adjacent to the infarct, but significant decreases were also seen in more remote brain areas. The observed inability to activate the particular cortical circuit testifies to the posttraumatic circuit dysfunction [32, 33]. In experiments designed specially to test plasticity after stroke in peri-infarct cortex, we examined plastic changes in the cortical representation of vibrissae, induced by sensory manipulation. Of the five rows of facial vibrissae, one was left intact and the others were shaved. In rats with unilateral focal photothrombotic stroke neighboring the whisker representation in the barrel cortex, we have observed (with 2DG autoradiography) the impairment of sensory deprivation-induced plasticity of functional vibrissae representation [34, 35] (Figure 2). This effect was replicated on mice with photothrombotic stroke (Liguz-Lecznar, unpublished data). Similarly, Greifzu and colleagues observed that a photothrombotic lesion outside the visual cortex prevented visual plasticity in mice. One week after stroke and monocular deprivation, the animals showed neither improvement of visual acuity and contrast sensitivity of the opened eye nor an ocular dominance shift toward this eye in the lesioned hemisphere [36]. Thus both in somatosensory cortex and in visual cortex no facilitating effect of stroke upon plasticity was observed. On the contrary, the effects of stroke were detrimental for experience-dependent plastic changes. In human, Cramer and Seitz [37] have used fMRI and measurements of blood oxygenation level-dependent (BOLD) method to show that the activation of peri-infarct tissue is reduced in patients with stroke compared with healthy controls.

Those observations seemed to be inconsistent with some results of receptor binding and electrophysiological studies that reported the increase of NMDA receptor binding [38], reduced inhibition [6, 39], and increased long-term potentiation in the surround of experimentally induced focal cortical infarction [40], which were in line with the poststroke plasticity facilitation concept. The discrepancies may be linked to the metabolic and physiological events taking place shortly after stroke. Edema, spreading depression, and inhibition of ionic pumps that accompany the first poststroke period are unlikely to support plastic changes [41]. Moreover, for at least several days after MCAO stroke in mice a protein synthesis impairment was observed [42], which can disturb the translation higher brain activity and long-term potentiation into the durable synaptic modification underlying plasticity of cortical representations. Also plasticity-limiting factors, such as outgrowth inhibitors (MAG, NoGO), extracellular matrix proteins (tenascin, chondroitin sulfate proteoglycans), and matrix metalloprotienases (MMPs) are triggered by brain injury and experimental stroke [43–45]. In our experiments the injection of the broad-spectrum MMPs inhibitor immediately before ischemia prevented the poststroke impairment of use-dependent plasticity in somatosensory cortex of mice [46] (Figure 2).

Deliberating the issue of plasticity after brain injury it has to be emphasized that mechanisms of injury in brain trauma and those observed in ischemia differ substantially regarding inflammation range and distribution, cell death dynamics, and free radical damage. Thus, the tissue environments induced by those two pathologies will generate distinct regions permissive for axonal sprouting and regeneration [24]. As we have shown, use-dependent plasticity is not facilitated by the stroke. On the contrary, stroke triggers a cascade of changes which may prevent the normal development of the plastic change. One of them may be the inflammatory reaction.

In rats and mice a plastic change in functional cortical somatosensory representation can be evoked by trimming all the rows of vibrissae except one. The cortical representation of this spared row of whiskers enlarges rapidly over neighboring cortex deprived of sensory input. We have described the impairment of experience-dependent plasticity in somatosensory cortex neighboring the infarct in mouse and rat. Plasticity was induced using deprivation-based protocol, in which all but one row of whiskers were trimmed unilaterally. Spared whiskers were contralateral to infarct. Deprivation-induced plasticity that started immediately after stroke did not evoke the expansion of spared whiskers representation (red color on the scheme) which was visible in control mice and rats after 7 and 30 days of deprivation, respectively.

In mice with stroke, single preischemic injection of matrix metalloproteinases inhibitor (FN-439) prevented the stroke-induced plasticity impairment. The enlargement of spared row of whiskers representation was similar to control animals [46].

In rats with ischemic stroke, daily injections of ibuprofen (nonselective cyclooxygenases inhibitor) both doses (10 and 20 mg/kg) partially reestablished the poststroke plasticity. Lower-dose treatment resulted in increase of spared row representation but however did not differ from stroke group. Injection of higher ibuprofen dose had stronger effect ad resulted in significantly higher expansion of spared row representation. Prorogation of deprivation onset in relation to stroke has similar effect on ibuprofen treatment. After one-week delay spared row representation did not differ significantly from rats deprived immediately after ischemia. Postponement of the deprivation onset to 28 days after stroke resulted in significantly larger expansion of spared row representation than in stroke group. However, it was smaller than in healthy animals [35].

## 3. Inflammation a Friend or an Enemy?

Stroke is known to be associated with inflammation. It is followed by acute and prolonged inflammatory response including the activation of glial cells, production of inflammatory cytokines, and infiltration of monocytes into the brain. It has been shown that these ischemic events contribute to brain injury, but, on the other hand, inflammatory cells can participate in tissue remodeling following brain damage [47]. The matter of detrimental versus beneficial influence of inflammatory response on brain function and recovery after stroke is extensively discussed [48]. There is abundant evidence that poststroke immune response embraces the releasing of number of destructive mediators including proinflammatory cytokines, matrix metalloproteinases, and reactive oxygen species [49], and many studies have reported beneficial effects of immunosuppressive manipulations on the stroke outcome (Table 1). 

The treatment with a tetracyclic antibiotic minocycline was shown to be successful neuroprotective agent. Recent paper by Liebigt et al. [50] has shown that, in rats, poststroke application of minocycline and indomethacin, combined with rehabilitative training, produces improved functional recovery compared to training alone. Through an anti-inflammatory action, reduction of microglial activation, matrix metalloproteinase activity, and nitric oxide production, minocycline was shown to reduce the infarct size and glutamate-induced cell death as well as to improve the functional recovery after stroke [51–53]. Several recent studies have reported improved functional outcome in minocycline-treated patients and suggested that it could be used with tissue plasminogen activator in treatment of acute ischemic stroke [54–56]. The therapy using sphingosine 1-phosphate receptor agonist (FTY720), which decreases the number of infiltrating T lymphocytes, was able to reduce the infarct volume, rescue neuronal death, and improve neurological score after MCAO in rats [57]. One of the recent therapies for experimental stroke has been intravenous immunoglobulin (IVIG) treatment which has the potential to inhibit multiple components of inflammation. The administration of IVIG to mice subjected to experimental MCAO stroke almost entirely eliminated mortality and reduced the amount of brain damage [58]. More precise pharmacological tools allow to target and inhibit specific factors in the complement cascade which influence the adhesion molecule upregulation, neutrophil chemotaxis, platelet activation, and generation of ROS, and it was shown that inhibition of specific complement components prevented tissue injury after stroke and reduced the infarct volume [59].

Other type of poststroke interventions comprises the anticytokine strategies, since experimental data indicate strongly that in animals and humans levels of several inflammatory cytokines, that is, IL-1, IL-6, or TNF-*α* are associated with the stroke severity and that the mitigation of inflammatory response attenuates the poststroke tissue damage [60–62]. IL-1 is not directly toxic to healthy neurons but contributes to neuronal death indirectly through actions on astrocytes and brain endothelial cells. It activates astrocytes leading to production of other inflammatory mediators like tumor necrosis factor alpha (TNF-*α*). The neuromodulatory effect of IL-1 is dose dependent: lower concentrations induce depolarization and higher hyperpolarization leading to the inhibition of synaptic transmission. IL-1 regulates phosphorylation of NMDA receptor and calcium influx and mediates excitotoxicity [63]. Since IL-1 appears to play little if any role in normal brain function, the IL-1 cytokine system seems to be particularly attractive therapeutic target in stroke. Applying interleukin-1 receptor antagonist in animal stroke models Loddick and Rothwell achieved neuroprotective effect in cerebral ischemia in rat [64]. Also IL-1 deficiency leads to reduced ischemic injury [65]. Moreover, it was shown that a single subcutaneous injection of IL-1RA was enough to reduce damage caused by MCAO in rats by 33%. IL-1RA penetrated brain tissue exclusively in areas of blood-brain barrier, rapidly reached salvageable brain tissue, and was able to confer its protective actions both peripherally and centrally [66].

TNF-*α* can be synthesized in CNS by resident macrophages, astrocytes, and microglia and is one of the central mediators of tissue inflammation that has been implicated in the pathogenesis of many neurological conditions. The activation of its receptor initiates signals leading to neuronal apoptosis. This cytokine seems to be proinflammatory during the acute phase of CNS inflammatory responses, but immunosuppressive during the chronic phase. TNF*α* stimulates expression of IL-1 and can induce also IL-6 [67]. Intraventricular injection of TNF-*α* enlarged infarct volume and brain edema after MCA occlusion in rats, whereas the injection of antibodies TNF-*α* reduced brain injury [62]. It was also shown that after MCAO mice deficient in TNF or functional CD95L, which belongs to the tumor necrosis factor (TNF) protein family, are protected against brain ischemia having reduced neuronal death and smaller degree of locomotor impairment [68]. Treatment with recombinant human TNF 55 kDa receptor reduced tissue damage and improved neurological symptoms after stroke in rats [69]. 

Interleukin 6 is a cytokine that controls an inflammatory response between blood cells, vascular endothelium, and brain parenchyma and can induce the synthesis of some chemokines and cell adhesion molecules that, together with blood-brain barrier leakage, can enable leukocyte infiltration. In the brain parenchyma, IL-6 activates gliosis and leukocyte activation [70]. Many investigators reported the association between IL-6 and early poststroke neurological worsening, proving that the level of IL-6 in plasma was highly correlated with infarct volume and positive association between IL-6 and the strength of the acute-phase response, which is considered to be a predictor of poor short-term clinical outcome [61, 71, 72].

In order to establish if the inflammatory processes are behind the impairment of plasticity observed after stroke, we examined the effects of chronic administration of ibuprofen, a nonselective inhibitor of cyclooxygenase 2 (an enzyme involved in postischemic tissue damage), on poststroke experience-dependent plasticity in somatosensory cortex. While in untreated rats photothrombotic stroke impaired this type of plasticity, in animals with daily doses of ibuprofen, we observed reestablishment of the modification of vibrissal cortical representation [35]. We also demonstrated that the level of cyclooxygenase 2, elevated after stroke, was reduced by the ibuprofen treatment. However, no neuroprotective effects were observed: the extent of stroke-induced damage was unchanged. Preliminary data of our experiments showed, moreover, that administration of soluble receptor of TNF-*α* after focal ischemia in mice has beneficial effect on functional plasticity in somatosensory cortex (Liguz-Lecznar, unpublished data). Ibuprofen treatment was also shown to be effective in restoration of the enhancement of vision after monocular deprivation that was abolished in stroked mice [36]. Thus, lowering of inflammatory reaction after stroke makes it possible to rescue cortical plasticity. Overcoming postischemic inflammation may be an important part of treatment leading to functional recovery. 

However, there is also evidence that contradicts the beneficial effects of immunosuppression [73], proving that the effect of particular components of the inflammatory cascade can be beneficial depending on the stage of tissue injury, the magnitude of the response, and whether the inflammatory component also activates neuroprotective pathways [74–76]. Development of therapeutic strategies based on the inhibition of TNF-*α* activity is complicated by its dual role. In mice that lacked both receptors for TNF-*α*: TNFR1 and TNFR2 which have been subjected to MCAO the infarct area and oxidative stress were larger than in wild-type controls, suggesting the cytoprotective role of TNF [74]. Also IL-6 as a pleiotropic mediator can potentially exert detrimental or beneficial effects following ischemia, as the administration of antimouse IL-6 receptor monoclonal antibody or knocking out IL-6 gene increased infarct size [70, 77]. Moreover, chronic injection of recombinant IL-6 into the lateral ventricle of prevented the postischemic learning disabilities and delayed neuronal loss and in other study reduced ischemic brain damage after MCAO [70].

## 4. Conclusions

Recovery after stroke is complex phenomenon. Many interventions have been developed to support the poststroke recovery and an increasing number of randomized controlled trials and systematic reviews are in progress. Spontaneous neuroplasticity triggered after stroke by behavioral demand of coordinated movements and the need for cognitive control gradually reorganizes brain connections. Injury causes the activation of numerous factors that impede plasticity, among them are several chemokines and cytokines involved in inflammatory reaction. Treatments inhibiting cyclooxygenases enhance poststroke plasticity. However, some elements of inflammatory cascade can improve recovery. Since postischemic inflammation is associated not only with ischemic damage but also with the repair of injured brain tissue, the most important aspect of therapies targeting the immune system will be to regulate the balance between the neurotoxic and neuroprotective effects of inflammatory state components. 

## Figures and Tables

**Table 1:** Examples of anti-inflammatory strategies effective in experimental stroke.

Target mediator in inflammatory cascade	Therapeutic intervention	Outcome	Literature examples
General inflammation	Minocycline	Attenuation of ischemic deficits are inhibition of apoptotic neuronal cell death after MCAO. Better outcome from stroke in patients	[54–56, 78]
	Intravenous immunoglobulin therapy	Elimination of mortality and reduction of brain damage after MCAO in mice	[58, 59]

Complement inhibition	Cobra venom factor (CVF)	Reduced infarct and atrophy are improved clinical outcome	[79, 80]
	*C1 inhibition*: C1-INH	Neuroprotection	[81]
	*C3 inhibition*: sCR1, C3 KO	Reduced infarct volume and neurological deficit score	[82, 83]
	*C5 inhibition*: C5 KO	Improvement of functional outcome, reduced brain damage	[58]

Leukocytes	Neutrophil inhibitory factor	Neuroprotection after focal ischemia in rats and reduction of the number of infiltrated neutrophils and infarct volume	[84, 85]

Lymphocytes T	Sphingosine 1-phosphate receptor agonist FTY720 (Fingolimod)	Reduction of infarct volume and cell death and improvement of neurological score after MCAO in rats	[57, 86, 87]

Prostaglandins	*Cyclooxygenase pathway:* Ibuprofen	Restoration of plasticity in visual and somatosensory cortex after photothrombotic stroke in mice without the neuroprotective effect	[35, 36]
	COX-2 KO	Reduction in the brain injury after MCAO in mice	[88]

Cytokines	*IL-1: *		
	IL-1 receptor antagonist	Administration of human interleukin-1 receptor antagonist reduced damage caused by MCAO in rats	[64, 66]
	IL-1 KO	70% reduction of infarct volume after MCAO	[65, 89, 90]
	IL-1R1 null mice	reduced brain damage and increased neuronal survival after ligation of right common carotid artery in mice	[91]
	*TNF*α*: *		
	Anti-TNF antibodies	Blocking endogenous TNF-alpha reduced focal ischemic brain injury in mice and rats after MCAO	[62, 92]
	TNF decoy receptor	Reduction of stroke volume, neural deficits, extent of microglial cell activation, and apoptotic cell death after MCAO in mice	[93, 94]
	Soluble TNF*α* receptor (TNFbp)	Reduction of infarct size after MCAO in mice	[75]

## References

[1] Dirnagl U, Iadecola C, Moskowitz MA (1999). Pathobiology of ischaemic stroke: an integrated view. *Trends in Neurosciences*.

[2] Lo EH, Dalkara T, Moskowitz MA (2003). Mechanisms, challenges and opportunities in stroke. *Nature Reviews Neuroscience*.

[3] Candelario-Jalil E (2009). Injury and repair mechanisms in ischemic stroke: considerations for the development of novel neurotherapeutics. *Current Opinion in Investigational Drugs*.

[4] Johansson BB (2000). Brain plasticity and stroke rehabilitation: the Willis lecture. *Stroke*.

[5] Carmichael ST (2003). Plasticity of cortical projections after stroke. *Neuroscientist*.

[6] Duncan PW, Jorgensen HS, Wade DT (2000). Outcome measures in acute stroke trials: a systematic review and some recommendations to improve practice. *Stroke*.

[7] Duncan PW (2002). Stroke recovery and rehabilitation research. *Journal of Rehabilitation Research and Development*.

[8] Studenski S, Duncan PW, Perera S, Reker D, Lai SM, Richards L (2005). Daily functioning and quality of life in a randomized controlled trial of therapeutic exercise for subacute stroke survivors. *Stroke*.

[9] Jablonka JA, Burnat K, Witte OW, Kossut M (2010). Remapping of the somatosensory cortex after a photothrombotic stroke: dynamics of the compensatory reorganization. *Neuroscience*.

[10] Wieloch T, Nikolich K (2006). Mechanisms of neural plasticity following brain injury. *Current Opinion in Neurobiology*.

[11] Nudo RJ, Wise BM, SiFuentes F, Milliken GW (1996). Neural substrates for the effects of rehabilitative training on motor recovery after ischemic infarct. *Science*.

[12] Bütefisch CM (2004). Plasticity in the human cerebral cortex: lessons from the normal brain and from stroke. *Neuroscientist*.

[13] Murphy TH, Corbett D (2009). Plasticity during stroke recovery: from synapse to behaviour. *Nature Reviews Neuroscience*.

[14] Carmichael ST, Wei L, Rovainen CM, Woolsey TA (2001). New patterns of intracortical projections after focal cortical stroke. *Neurobiology of Disease*.

[15] Li S, Overman JJ, Katsman D (2010). An age-related sprouting transcriptome provides molecular control of axonal sprouting after stroke. *Nature Neuroscience*.

[16] Carmichael ST (2005). Rodent models of focal stroke: size, mechanism, and purpose. *NeuroRx*.

[17] Brown CE, Wong C, Murphy TH (2008). Rapid morphologic plasticity of peri-infarct dendritic spines after focal ischemic stroke. *Stroke*.

[18] Cramer SC (2000). Stroke recovery: how the computer reprograms itself. Neuronal plasticity: the key to stroke recovery. Kananskis, Alberta, Canada, 19-22 March 2000. *Molecular Medicine Today*.

[19] Mostany R, Chowdhury TG, Johnston DG, Portonovo SA, Carmichael ST, Portera-Cailliau C (2010). Local hemodynamics dictate long-term dendritic plasticity in peri-infarct cortex. *Journal of Neuroscience*.

[20] Brown CE, Aminoltejari K, Erb H, Winship IR, Murphy TH (2009). In vivo voltage-sensitive dye imaging in adult mice reveals that somatosensory maps lost to stroke are replaced over weeks by new structural and functional circuits with prolonged modes of activation within both the peri-infarct zone and distant sites. *Journal of Neuroscience*.

[21] Jones RD, Donaldson IM, Parkin PJ (1989). Impairment and recovery of ipsilateral sensory-motor function following unilateral cerebral infarction. *Brain*.

[22] Witte OW (1998). Lesion-induced plasticity as a potential mechanism for recovery and rehabilitative training. *Current Opinion in Neurology*.

[23] Jones TA, Chu CJ, Grande LA, Gregory AD (1999). Motor skills training enhances lesion-induced structural plasticity in the motor cortex of adult rats. *Journal of Neuroscience*.

[24] Carmichael ST (2006). Cellular and molecular mechanisms of neural repair after stroke: making waves. *Annals of Neurology*.

[25] van Meer MP, Otte WM, van der Marel K (2012). Extent of bilateral neuronal network reorganization and functional recovery in relation to stroke severity. *The Journal of Neuroscience*.

[26] Sterr A, Shan Shen, Szameitat AJ, Herron KA (2010). The role of corticospinal tract damage in chronic motor recovery and neurorehabilitation: a pilot study. *Neurorehabilitation and Neural Repair*.

[27] Qiu M, Darling WG, Morecraft RJ, Ni CC, Rajendra J, Butler AJ (2011). White matter integrity is a stronger predictor of motor function than BOLD response in patients with stroke. *Neurorehabilitation and Neural Repair*.

[28] Borich MR, Mang C, Boyd LA (2012). Both projection and commissural pathways are disrupted in individuals with chronic stroke: investigating microstructural white matter correlates of motor recovery. *BMC Neuroscience*.

[29] Schlaug G, Marchina S, Norton A (2009). Evidence for plasticity in white-matter tracts of patients with chronic broca’s aphasia undergoing intense intonation-based speech therapy. *Annals of the New York Academy of Sciences*.

[30] Bush TG, Puvanachandra N, Horner CH (1999). Leukocyte infiltration, neuronal degeneration, and neurite outgrowth after ablation of scar-forming, reactive astrocytes in adult transgenic mice. *Neuron*.

[31] Karetko-Sysa M, Skangiel-Kramska J, Nowicka D (2011). Disturbance of perineuronal nets in the perilesional area after photothrombosis is not associated with neuronal death. *Experimental Neurology*.

[32] Dietrich WD, Alonso O, Busto R, Ginsberg MD (1994). Widespread metabolic depression and reduced somatosensory circuit activation following traumatic brain injury in rats. *Journal of Neurotrauma*.

[33] Passineau MJ, Zhao W, Busto R (2000). Chronic metabolic sequelae of traumatic brain injury: prolonged suppression of somatosensory activation. *American Journal of Physiology*.

[34] Jablonka J, Kossut M (2006). Focal stroke in the barrel cortex of rats enhances ipsilateral response to vibrissal input. *Acta Neurobiologiae Experimentalis*.

[35] Jablonka JA, Kossut M, Witte OW, Liguz-Lecznar M (2012). Experience-dependent brain plasticity after stroke: effect of ibuprofen and poststroke delay. *European Journal of Neuroscience*.

[36] Greifzu F, Schmidt S, Schmidt KF, Kreikemeier K, Witte OW (2011). Global impairment and therapeutic restoration of visual plasticity mechanisms after a localized cortical stroke. *Proceedings of the National Academy of Sciences of the United States of America*.

[37] Cramer SC, Seitz RJ (2008). Imaging functional recovery from stroke. *Handbook of Clinical Neurology*.

[38] Que M, Schiene K, Witte OW, Zilles K (1999). Widespread up-regulation of N-methyl-D-aspartate receptors after focal photothrombotic lesion in rat brain. *Neuroscience Letters*.

[39] Neumann-Haefelin T, Witte OW (2000). Periinfarct and remote excitability changes after transient middle cerebral artery occlusion. *Journal of Cerebral Blood Flow and Metabolism*.

[40] Hagemann G, Redecker C, Neumann-Haefelin T, Freund HJ, Witte OW (1998). Increased long-term potentiation in the surround of experimentally induced focal cortical infarction. *Annals of Neurology*.

[41] Hossmann KA (2000). The hypoxic brain: insights from ischemia research. *Advances in Experimental Medicine and Biology*.

[42] Hata R, Maeda K, Hermann D, Mies G, Hossmann KA (2000). Evolution of brain infarction after transient focal cerebral ischemia in mice. *Journal of Cerebral Blood Flow and Metabolism*.

[43] Cheatwood JL, Emerick AJ, Schwab ME, Kartje GL (2008). Nogo-A expression after focal ischemic stroke in the adult rat. *Stroke*.

[44] Li S, Carmichael ST (2006). Growth-associated gene and protein expression in the region of axonal sprouting in the aged brain after stroke. *Neurobiology of Disease*.

[45] Kaczmarek L (2012). MMP-9 inhibitors in the brain: can old bullets shoot new targets?. *Current Pharmaceutical Designs*.

[46] Cybulska-Klosowicz A, Liguz-Lecznar M, Nowicka D, Ziemka-Nalecz M, Kossut M, Skangiel-Kramska J (2011). Matrix metalloproteinase inhibition counteracts impairment of cortical experience-dependent plasticity after photothrombotic stroke. *European Journal of Neuroscience*.

[47] Kriz J (2006). Inflammation in ischemic brain injury: timing is important. *Critical Reviews in Neurobiology*.

[48] Macrez R, Ali C, Toutirais O (2011). Stroke and the immune system: from pathophysiology to new therapeutic strategies. *The Lancet Neurology*.

[49] Wang Q, Tang XN, Yenari MA (2007). The inflammatory response in stroke. *Journal of Neuroimmunology*.

[50] Liebigt S, Schlegel N, Oberland J, Witte OW, Redecker C, Keiner S (2012). Effects of rehabilitative training and anti-inflammatory treatment on functional recovery and cellular reorganization following stroke. *Experimental Neurology*.

[51] Morimoto N, Shimazawa M, Yamashima T, Nagai H, Hara H (2005). Minocycline inhibits oxidative stress and decreases in vitro and in vivo ischemic neuronal damage. *Brain Research*.

[52] Weng YC, Kriz J (2007). Differential neuroprotective effects of a minocycline-based drug cocktail in transient and permanent focal cerebral ischemia. *Experimental Neurology*.

[53] Emsley HCA, Smith CJ, Tyrrell PJ, Hopkins SJ (2008). Inflammation in acute ischemic stroke and its relevance to stroke critical care. *Neurocritical Care*.

[54] Padma Srivastava MV, Bhasin A, Bhatia R (2012). Efficacy of minocycline in acute ischemic stroke: a single-blinded, placebo-controlled trial. *Neurology India*.

[55] Fagan SC, Cronic LE, Hess DC (2011). Minocycline development for acute ischemic stroke. *Translational Stroke Research*.

[56] Fagan SC, Waller JL, Nichols FT (2010). Minocycline to improve neurologic outcome in stroke (MINOS): a dose-finding study. *Stroke*.

[57] Hasegawa Y, Suzuki H, Sozen T, Rolland W, Zhang JH (2010). Activation of sphingosine 1-phosphate receptor-1 by FTY720 is neuroprotective after ischemic stroke in rats. *Stroke*.

[58] Arumugam TV, Tang SC, Lathia JD (2007). Intravenous immunoglobulin (IVIG) protects the brain against experimental stroke by preventing complement-mediated neuronal cell death. *Proceedings of the National Academy of Sciences of the United States of America*.

[59] Arumugam TV, Woodruff TM, Lathia JD, Selvaraj PK, Mattson MP, Taylor SM (2009). Neuroprotection in stroke by complement inhibition and immunoglobulin therapy. *Neuroscience*.

[60] Beamer NB, Coull BM, Clark WM, Hazel JS, Silberger JR (1995). Interleukin-6 and interleukin-1 receptor antagonist in acute stroke. *Annals of Neurology*.

[61] Vila N, Castillo J, Dávalos A, Chamorro A (2000). Proinflammatory cytokines and early neurological worsening in ischemic stroke. *Stroke*.

[62] Barone FC, Arvin B, White RF (1997). Tumor necrosis factor-*α*: a mediator of focal ischemic brain injury. *Stroke*.

[63] Denes A, Pinteaux E, Rothwell NJ, Allan SM (2011). Interleukin-1 and stroke: biomarker, harbinger of damage, and therapeutic target. *Cerebrovascular Diseases*.

[64] Loddick SA, Rothwell NJ (1996). Neuroprotective effects of human recombinant interleukin-1 receptor antagonist in focal cerebral ischaemia in the rat. *Journal of Cerebral Blood Flow and Metabolism*.

[65] Vexler ZS, Tang XN, Yenari MA (2006). Inflammation in adult and neonatal stroke. *Clinical Neuroscience Research*.

[66] Greenhalgh AD, Galea J, Dénes A, Tyrrell PJ, Rothwell NJ (2010). Rapid brain penetration of interleukin-1 receptor antagonist in rat cerebral ischaemia: pharmacokinetics, distribution, protection. *British Journal of Pharmacology*.

[67] Lucas SM, Rothwell NJ, Gibson RM (2006). The role of inflammation in CNS injury and disease. *British Journal of Pharmacology*.

[68] Martin-Villalba A, Hahne M, Kleber S (2001). Therapeutic neutralization of CD95-ligand and TNF attenuates brain damage in stroke. *Cell Death and Differentiation*.

[69] Sirén AL, McCarron R, Wang L (2001). Proinflammatory cytokine expression contributes to brain injury provoked by chronic monocyte activation. *Molecular Medicine*.

[70] Suzuki S, Tanaka K, Suzuki N (2009). Ambivalent aspects of interleukin-6 in cerebral ischemia: inflammatory versus neurotrophic aspects. *Journal of Cerebral Blood Flow and Metabolism*.

[71] Smith CJ, Emsley HCA, Gavin CM (2004). Peak plasma interleukin-6 and other peripheral markers of inflammation in the first week of ischaemic stroke correlate with brain infarct volume, stroke severity and long-term outcome. *BMC Neurology*.

[72] Waje-Andreassen U, Kråkenes J, Ulvestad E (2005). IL-6: an early marker for outcome in acute ischemic stroke. *Acta Neurologica Scandinavica*.

[73] Krams M, Lees KR, Hacke W, Grieve AP, Orgogozo JM, Ford GA (2003). Acute Stroke Therapy by Inhibition of Neutrophils (ASTIN): an adaptive dose-response study of UK-279,276 in acute ischemic stroke. *Stroke*.

[74] Bruce AJ, Boling W, Kindy MS (1996). Altered neuronal and microglial responses to excitotoxic and ischemic brain injury in mice lacking TNF receptors. *Nature Medicine*.

[75] Nawashiro H, Martin D, Hallenbeck JM (1997). Neuroprotective effects of TNF binding protein in focal cerebral ischemia. *Brain Research*.

[76] Zhang W, Stanimirovic D (2002). Current and future therapeutic strategies to target inflammation in stroke. *Curr Drug Targets Inflamm Allergy*.

[77] Yamashita T, Sawamoto K, Suzuki S (2005). Blockade of interleukin-6 signaling aggravates ischemic cerebral damage in mice: possible involvement of Stat3 activation in the protection of neurons. *Journal of Neurochemistry*.

[78] Xu L, Fagan SC, Waller JL (2004). Low dose intravenous minocycline is neuroprotective after middle cerebral artery occlusion-reperfusion in rats. *BMC Neurology*.

[79] Figueroa E, Gordon LE, Feldhoff PW, Lassiter HA (2005). The administration of cobra venom factor reduces post-ischemic cerebral injury in adult and neonatal rats. *Neuroscience Letters*.

[80] Vasthare US, Barone FC, Sarau HM (1998). Complement depletion improves neurological function in cerebral ischemia. *Brain Research Bulletin*.

[81] Heydenreich N, Nolte MW, Gob E (2012). C1-inhibitor protects from brain ischemia-reperfusion injury by combined antiinflammatory and antithrombotic mechanisms. *Stroke*.

[82] Ducruet AF, Hassid BG, MacK WJ (2008). C3a receptor modulation of granulocyte infiltration after murine focal cerebral ischemia is reperfusion dependent. *Journal of Cerebral Blood Flow and Metabolism*.

[83] Mocco J, Mack WJ, Ducruet AF (2006). Complement component C3 mediates inflammatory injury following focal cerebral ischemia. *Circulation Research*.

[84] Jiang N, Chopp M, Chahwala S (1998). Neutrophil inhibitory factor treatment of focal cerebral ischemia in the rat. *Brain Research*.

[85] Jiang N, Moyle M, Soule HR, Rote WE, Chopp M (1995). Neutrophil inhibitory factor is neuroprotective after focal ischemia in rats. *Annals of Neurology*.

[86] Czech B, Pfeilschifter W, Mazaheri-Omrani N (2009). The immunomodulatory sphingosine 1-phosphate analog FTY720 reduces lesion size and improves neurological outcome in a mouse model of cerebral ischemia. *Biochemical and Biophysical Research Communications*.

[87] Wei Y, Yemisci M, Kim HH (2011). Fingolimod provides long-term protection in rodent models of cerebral ischemia. *Annals of Neurology*.

[88] Iadecola C, Niwa K, Nogawa S (2001). Reduced susceptibility to ischemic brain injury and N-methyl-D-aspartate-mediated neurotoxicity in cyclooxygenase-2-deficient mice. *Proceedings of the National Academy of Sciences of the United States of America*.

[89] Boutin H, LeFeuvre RA, Horai R, Asano M, Iwakura Y, Rothwell NJ (2001). Role of IL-1*α* and IL-1*β* in ischemic brain damage. *Journal of Neuroscience*.

[90] Touzani O, Boutin H, Lefeuvre R (2002). Interleukin-1 influences ischemic brain damage in the mouse independently of the interleukin-1 type I receptor. *Journal of Neuroscience*.

[91] Basu A, Lazovic J, Krady JK (2005). Interleukin-1 and the interleukin-1 type 1 receptor are essential for the progressive neurodegeneration that ensues subsequent to a mild hypoxic/ischemic injury. *Journal of Cerebral Blood Flow and Metabolism*.

[92] Yang GY, Gong C, Qin Z, Ye W, Mao Y, Bertz AL (1998). Inhibition of TNF*α* attenuates infarct volume and ICAM-1 expression in ischemic mouse brain. *NeuroReport*.

[93] Yepes M, Brown SAN, Moore EG, Smith EP, Lawrence DA, Winkles JA (2005). A soluble Fn14-Fc decoy receptor reduces infarct volume in a murine model of cerebral ischemia. *American Journal of Pathology*.

[94] Sumbria RK, Boado RJ, Pardridge WM (2012). Brain protection from stroke with intravenous TNF*α* decoy receptor-Trojan horse fusion protein. *Journal of Cerebral Blood Flow & Metabolism*.

